# Prognostic value of the metabolic score obtained via [^18^F]FDG PET/CT and a new prognostic staging system for gastric cancer

**DOI:** 10.1038/s41598-022-24877-0

**Published:** 2022-11-30

**Authors:** Sung Hoon Kim, Bong-Il Song, Hae Won Kim, Kyoung Sook Won, Young-Gil Son, Seung Wan Ryu, Yoo Na Kang

**Affiliations:** 1 Department of Nuclear Medicine, Keimyung University School of Medicine, Daegu, Korea; 2 Department of Nuclear Medicine, Yeungnam University Hospital, Daegu, Korea; 3 Department of Nuclear Medicine, Keimyung University Dongsan Hospital, Daegu, Korea; 4 Department of Surgery, Keimyung University Dongsan Hospital, Keimyung University School of Medicine, Daegu, Korea; 5 Department of Forensic Medicine, Kyungpook National University School of Medicine, Daegu, Korea

**Keywords:** Cancer imaging, Gastrointestinal cancer

## Abstract

We developed and validated a new staging system that includes metabolic information from pretreatment [^18^F]Fluorodeoxyglucose ([^18^F]FDG) positron emission tomography/computed tomography (PET/CT) for predicting disease-specific survival (DSS) in gastric cancer (GC) patients. Overall, 731 GC patients undergoing preoperative [^18^F]FDG PET/CT were enrolled and divided into the training (n = 543) and validation (n = 188) cohorts. A metabolic score (MS) was developed by combining the maximum standardized uptake value (SUVmax) of the primary tumor (T_SUVmax) and metastatic lymph node (N_SUVmax). A new staging system incorporating the MS and tumor-node-metastasis (TNM) stage was developed using conditional inference tree analysis. The MS was stratified as follows: score 1 (T_SUVmax ≤ 4.5 and N_SUVmax ≤ 1.9), score 2 (T_SUVmax > 4.5 and N_SUVmax ≤ 1.9), score 3 (T_SUVmax ≤ 4.5 and N_SUVmax > 1.9), and score 4 (T_SUVmax > 4.5 and N_SUVmax > 1.9) in the training cohort. The new staging system yielded five risk categories: category I (TNM I, II and MS 1), category II (TNM I, II and MS 2), category III (TNM I, II and MS ≥ 3), category IV (TNM III, IV and MS ≤ 3), and category V (TNM III, IV and MS 4) in the training cohort. DSS differed significantly between both staging systems; the new staging system showed better prognostic performance in both training and validation cohorts. The MS was an independent prognostic factor for DSS, and discriminatory power of the new staging system for DSS was better than that of the conventional TNM staging system alone.

## Introduction

Gastric cancer (GC) is the fifth most common cancer and the fourth leading cause of cancer-related deaths worldwide^1^. Although the mortality rate of GC patients has continuously decreased, the survival rate markedly varies among countries, and the prognosis remains poor^2,3^. For potentially resectable GC patients, a combined-modality treatment that includes surgery, perioperative chemotherapy, or chemoradiation therapy is recommended^4^. In recent years, new treatment options such as targeted agents or immunotherapy have become available for high-risk disease patients^5,6^. Accordingly, improving risk stratification for GC patients after surgery has become more important for predicting long-term survival outcomes and further therapeutic planning.

Currently, the tumor-node-metastasis (TNM) staging system developed by the American Joint Committee on Cancer (AJCC) is the most commonly used method for classifying and predicting GC prognosis^7^. However, the anatomy-based TNM staging has limited use as a predictive tool for assessing individual patient survival because it does not include several significant factors that affect prognosis owing to the need for simplicity and uniform application of the staging system^8^. Better prognostic models for the accurate prediction of survival outcomes and identification of patients with poor prognoses are needed for tailored treatment.

Positron emission tomography/computed tomography (PET/CT) with [^18^F]Fluorodeoxyglucose ([^18^F]FDG) has become a widely used method for staging, response evaluation, recurrence detection, and restaging of GC^9,10^. Despite the controversy regarding its routine use in GC patients owing to its unsatisfactory sensitivity for a primary tumor or lymph node (LN) involvement^11,12^, recent studies have demonstrated a prognostic value of preoperative [^18^F]FDG PET/CT for GC. [^18^F]FDG uptake by the primary tumor reflects biological aggressiveness, and the high specificity of PET/CT for detecting LN and occult metastases could be valuable in predicting prognosis based on metabolic information, although it does not provide exquisite anatomic details^9,13,14^.

Several studies have revealed that positive [^18^F]FDG uptake by primary gastric tumor is associated with inferior overall survival^15,16^ and [^18^F]FDG uptake by metastatic LN could be a surrogate prognostic marker^17^; however, there is no cooperative analysis study that uses both the metabolic activities of the primary tumor and metastatic LN for risk stratification in GC patients. Furthermore, no studies have attempted to develop a staging system for disease-specific survival (DSS) in GC patients using metabolic information from pretreatment [^18^F]FDG PET/CT.

Thus, this retrospective study aimed to investigate the prognostic impact of a metabolic score comprising the maximum standardized uptake value (SUVmax) of the primary tumor (T_SUVmax) and the metastatic LN (N_SUVmax) obtained via preoperative [^18^F]FDG PET/CT to predict DSS in GC patients. Furthermore, we assessed the additional prognostic value of an [^18^F]FDG PET/CT parameter for improving risk stratification. Finally, we developed a novel staging system using the metabolic score and compared its efficiency with that of conventional TNM staging for predicting DSS after curative surgery for GC.

## Results

### Patient characteristics

Among 731 patients included in the analysis, 90 (12.3%) died within a median follow-up period of 87.2 (range 1.8–109.2) months. The overall 3- and 5-year DSS rates were 90.8% and 88.2%, respectively. The median DSS were 88.8 (range 1.8–109.2) and 21.3 (range 1.9–93.1) months among survivors and nonsurvivors, respectively. The mean T_SUVmax and N_SUVmax were 3.4 ± 4.6 and 0.4 ± 1.8 in survivors and 6.7 ± 4.8 and 1.8 ± 3.6 in nonsurvivors, respectively.

The characteristics of the enrolled patients in the training cohort (n = 543) and validation cohort (n = 188) are summarized in Table 1. Regarding the TNM stage, 314 (57.8%) patients had stage I GC, 94 (17.3%) stage II GC, 132 (24.3%) stage III GC, and 3 (0.6%) stage IV GC in the training cohort. Meanwhile, 123 (65.4%) patients were the stage I, 36 (19.1%) were stage II, 28 (14.9%) were stage III and 1 (0.5%) were stage IV in the validation cohort. Three of the four stage IV GC patients presented with hepatic metastasis, and the other presented with a seeding mass in the sigmoid colon mesentery. These patients underwent radical gastrectomy with a curative aim accompanied by metastatic mass excision.

**Table 1:** Patient characteristics in the training and validation cohorts.

Characteristics	Training cohort, N (%) (N = 543)	Validation cohort, N (%) (N = 188)
**Sex**		
Male	330 (60.8%)	123 (65.4%)
Female	213 (39.2%)	65 (34.6%)
Age (years)	59.6 ± 12.0^a^	60.1 ± 11.8^a^
**Tumor location**		
Upper	111 (20.4%)	31 (16.5%)
Middle	98 (18.0%)	36 (19.1%)
Lower	301 (55.4%)	115 (61.2%)
Whole/multicentric	33 (6.1%)	6 (3.2%)
**Pathologic T stage**		
T1	298 (54.9%)	123 (65.4%)
T2	70 (12.9%)	15 (8.0%)
T3	69 (12.7%)	31 (16.5%)
T4	106 (19.5%)	19 (10.1%)
**Pathologic N stage**		
N0	333 (61.3%)	132 (70.2%)
N1	63 (11.6%)	23 (12.2%)
N2	51 (9.4%)	12 (6.4%)
N3	96 (17.7%)	21 (11.2%)
**8th AJCC TNM stage**		
I	314 (57.8%)	123 (65.4%)
II	94 (17.3%)	36 (19.1%)
III	132 (24.3%)	28 (14.9%)
IV	3 (0.6%)	1 (0.5%)
**WHO histopathologic subtype**		
Tubular adenocarcinoma	414 (76.3%)	138 (73.4%)
Signet ring cell	107 (19.7%)	41 (21.8%)
Mucinous	12 (2.2%)	4 (2.1%)
Others^b^	10 (1.8%)	5 (2.7%)
**Lauren histotype**		
Diffuse	291 (53.6%)	103 (54.8%)
Intestinal	243 (44.8%)	85 (45.2%)
Mixed	9 (1.7%)	0 (0%)
**Lymphovascular invasion**		
Positive	302 (55.8%)	103 (54.8%)
Negative	239 (44.2%)	85 (45.2%)
**Neural invasion**		
Positive	360 (66.4%)	147 (78.2%)
Negative	182 (33.6%)	41 (21.8%)
T_SUVmax	4.4 ± 4.6^a^	4.1 ± 4.0^a^
N_SUVmax	1.6 ± 2.0^a^	1.5 ± 1.6^a^
**Metabolic score**		
1	359 (66.1%)	131 (69.7%)
2	125 (23.0%)	38 (20.2%)
3	11 (2.0%)	5 (2.7%)
4	48 (8.9%)	14 (7.4%)

*T_SUVmax* maximum standardized uptake value of the primary tumor, *N_SUVmax* maximum standardized uptake value of the metastatic lymph node, *AJCC* American Joint Committee on Cancer, *TNM* tumor-node-metastasis.

^a^Data are presented as mean with standard deviation.

^b^Others contain papillary subtype and rare variants.

### Prognostic factors for DSS

**Figure 1:**
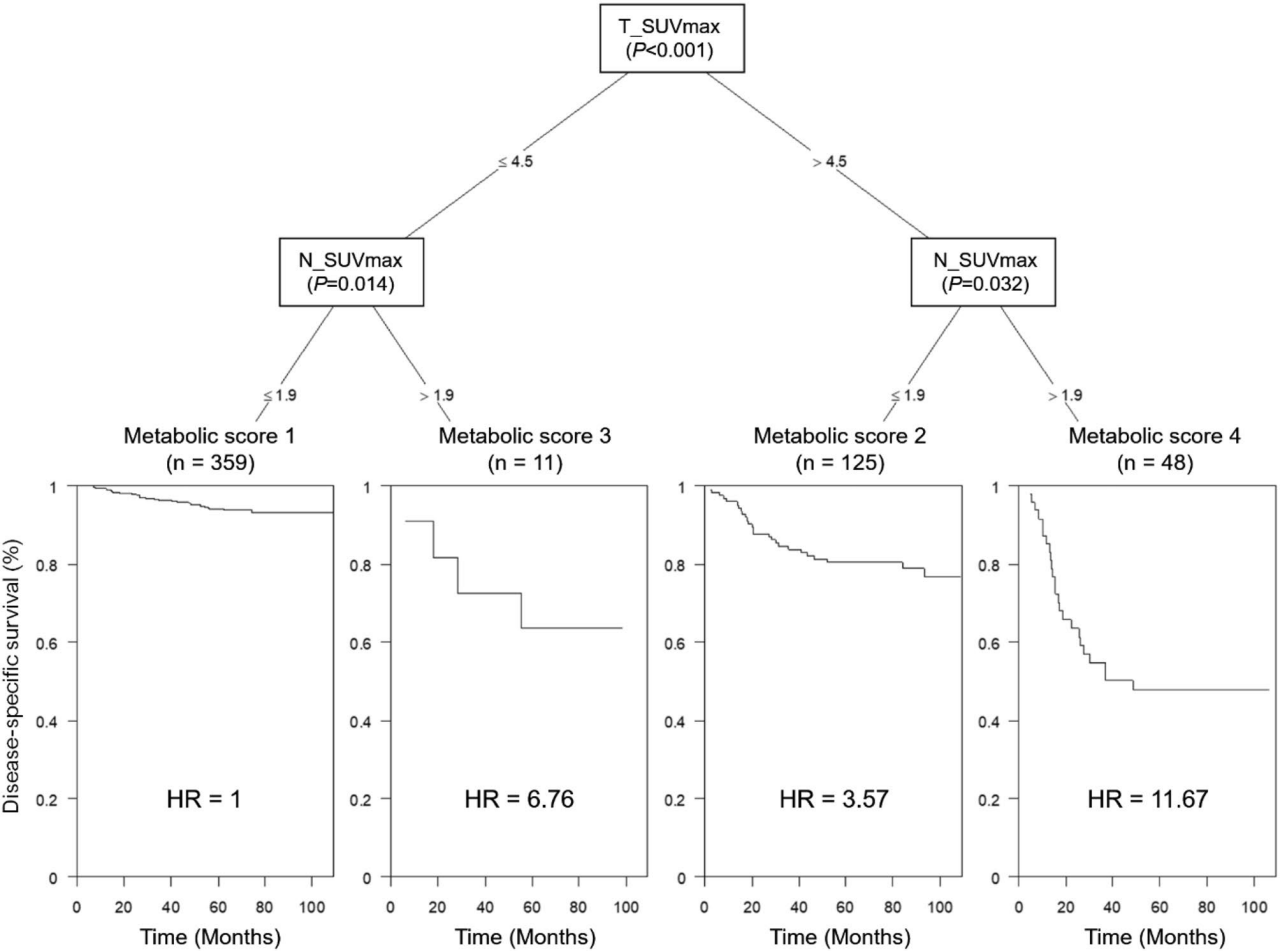
Tree-structured survival analyses for the metabolic score by combining T_SUVmax and N_SUVmax in the training cohort. The optimal cut-off values of T_SUVmax and N_SUVmax for the metabolic score determined using conditional inference trees analysis were 4.5 and 1.9, respectively. T_SUVmax = maximum standardized uptake value of primary tumor; N_SUVmax = maximum standardized uptake value of metastatic lymph node.

**Figure 2:**
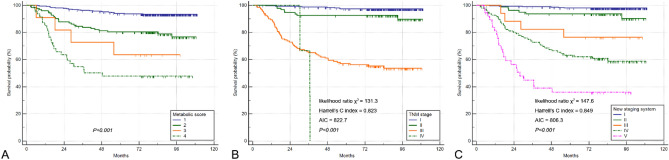
Cumulative DSS curves of the 543 gastric cancer patients according to the metabolic score (**A**), TNM stage (**B**), and new staging system (**C**) in the training cohort. *DSS* disease-specific survival, *TNM* tumor-node-metastasis.

We developed the metabolic score based on PET-derived variables, i.e. T_SUVmax and N_SUVmax, for predicting DSS using conditional inference trees (CTree) analysis in the training cohort. The optimal cut-off values of T_SUVmax and N_SUVmax for the metabolic score determined by CTree analysis were 4.5 and 1.9, respectively. The scores were as follows: metabolic score 1 (T_SUVmax ≤ 4.5 and N_SUVmax ≤ 1.9), metabolic score 2 (T_SUVmax > 4.5 and N_SUVmax ≤ 1.9), metabolic score 3 (T_SUVmax ≤ 4.5 and N_SUVmax > 1.9), and metabolic score 4 (T_SUVmax > 4.5 and N_SUVmax > 1.9). In total, 359 (66.1%), 125 (23.0%), 11 (2.0%), and 48 (8.9%) patients had metabolic scores of 1, 2, 3, and 4, respectively (Fig. 1). Kaplan–Meier analysis showed that a higher metabolic score was associated with a poorer DSS, and the log-rank test showed a significant difference in survival between the metabolic scores (*P* < 0.001) (Fig. 2A).

Univariate Cox proportional hazards regression analysis revealed that pathologic T (pT) stage, pathologic N (pN) stage, lymphovascular invasion, neural invasion, and the metabolic score were significantly associated with DSS in the training cohort (Table 2). In the multivariate analysis, pT stage (hazard ratio (HR) 1.69; 95% confidence interval (CI) 1.25–2.30; *P* < 0.001), pN stage (HR 1.69; 95% CI 1.29–2.22; *P* < 0.001), and the metabolic score (HR 1.28; 95% CI 1.06–1.56; *P* = 0.012) remained independent prognostic factors of DSS.

**Table 2:** Univariate and multivariate analyses of prognostic factors of disease-specific survival.

Variables	Univariate analysis		Multivariate analysis	
	HR (95% CI)	P value	HR (95% CI)	P value
Age, years (continuous)	1.01 (0.99–1.03)	0.375		
Sex (male vs female)	0.94 (0.59–1.49)	0.782		
Pathologic T stage (T1, T2, T3, T4)	2.88 (2.30–3.60)	< 0.001	1.69 (1.25–2.30)	< 0.001
Pathologic N stage (N0, N1, N2, N3)	2.88 (2.34–3.54)	< 0.001	1.69 (1.29–2.22)	< 0.001
WHO classification	1.11 (0.78–1.56)	0.568		
Lauren classification	0.71 (0.45–1.10)	0.123		
Location of tumor	1.21 (0.93–1.59)	0.160		
Lymphovascular invasion (negative vs positive)	8.82 (4.66–16.70)	< 0.001	1.56 (0.74–3.28)	0.238
Neural invasion (negative vs positive)	7.23 (4.31–12.15)	< 0.001	1.21 (0.66–2.24)	0.539
Metabolic score (1, 2, 3, 4)	2.22 (1.86–2.65)	< 0.001	1.28 (1.06–1.56)	0.012

*HR* hazard ratio, *CI* confidence interval.

### Construction of a new staging system incorporating the TNM stage and metabolic score

**Figure 3:**
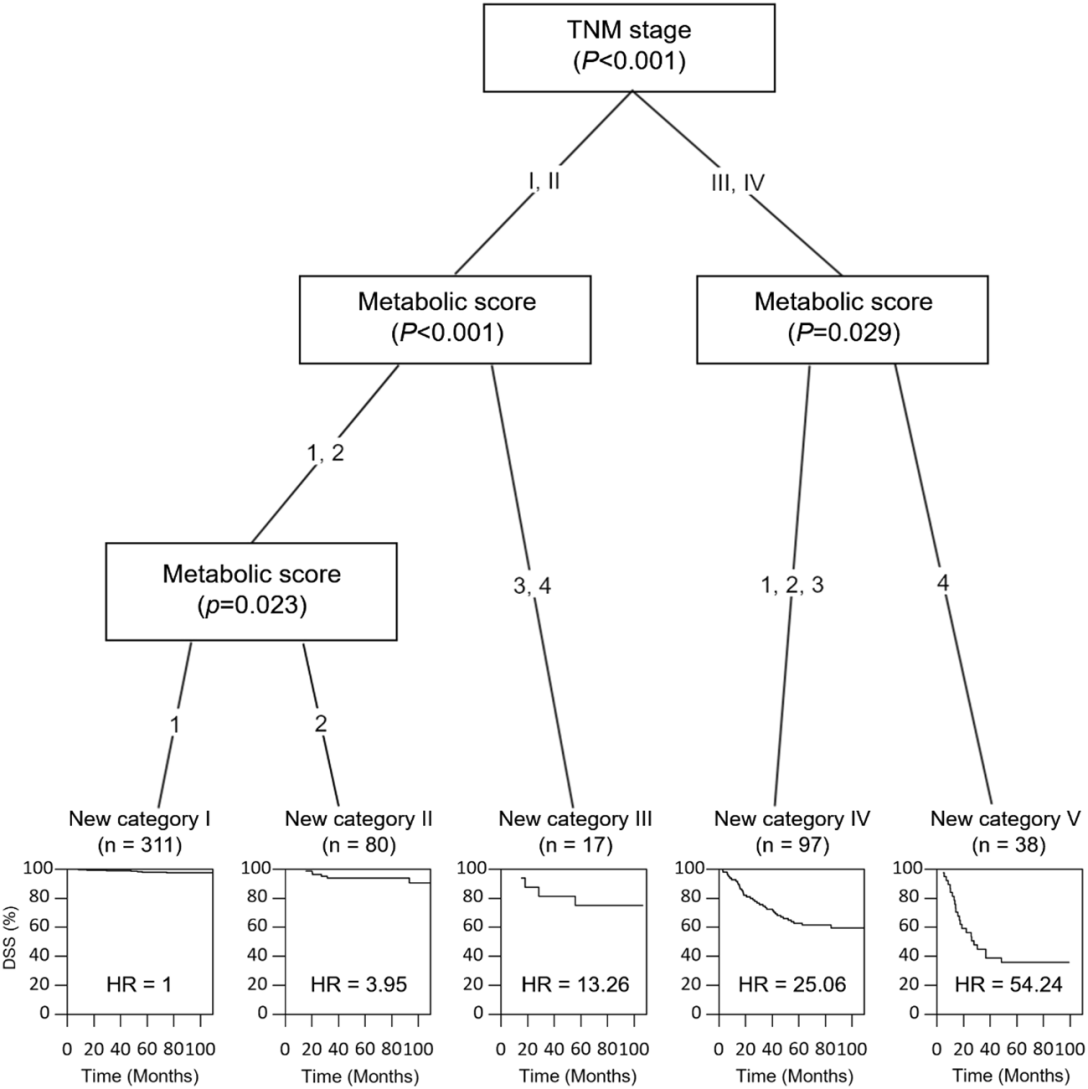
A new staging system using the metabolic score and TNM stage using tree-structured survival analyses. Five terminal risk groups (new staging system) were established in the training cohort. *TNM* tumor-node-metastasis.

We constructed a hierarchical prognostic model for predicting DSS. Because metabolic score was determined to be an independent prognostic factor, we combined the metabolic score with the TNM stage for the new staging system. Patients were categorized into five new risk groups based on the results of the CTree analysis in the training cohort (Fig. 3): category I (TNM stage I, II and metabolic score 1), category II (TNM stage I, II and metabolic score 2), category III (TNM stage I, II and metabolic score ≥ 3), category IV (TNM stage III, IV and metabolic score ≤ 3), and category V (TNM stage III, IV and metabolic score 4). Regarding stage distribution according to the new prognostic system, 311 (57.3%) patients were classified into category I; 80 (14.7%) into category II; 17 (3.1%) into category III; 97 (17.9%) into category IV; and 38 (7.0%) into category V (Table 3).

**Table 3:** Patient distribution according to the 8th AJCC TNM stage and new staging system.

8th AJCC TNM stage	New staging system					
	I	II	III	IV	V	
I	263	47	4	0	0	314
II	48	33	13	0	0	94
III	0	0	0	97	35	132
IV	0	0	0	0	3	3
	311	80	17	97	38	

*AJCC* American Joint Committee on Cancer, *TNM* tumor-node-metastasis.

### Comparison of prognostic performance between the TNM stage and new staging system

According to the TNM staging system, the DSS rates were 97.5% for stage I GC patients, 91.5% for stage II, 56.1% for stage III, and 0% for stage IV (*P* < 0.001) (Fig. 2B). In the new staging system, the DSS rates were 98.1% for category I, 92.5% for category II, 76.5% for category III, 60.8% for category IV, and 39.5% for category V (*P* < 0.001) (Fig. 2C). The calculated HRs for DSS (reference group: category I in the new staging system) increased in a stepwise manner (3.95 for category II, 13.26 for category III, 25.06 for category IV, and 54.24 for category V) in the training cohort.

Comparison of prognostic performance of the two prognostic models according to the χ^2^ likelihood ratio, Harrell’s C index, and Akaike information criterion (AIC) values showed that compared with the TNM stage, the new staging system had better homogeneity (χ^2^ likelihood ratio: 147.6 vs 131.3) and discriminatory capability (Harrell’s C index: 0.849 vs 0.823; AIC value: 806.3 vs 822.7) in the training cohort. This finding indicates that combining PET metabolic variables with the pathologic TNM stage could provide better prognostic stratification of GC patients.

### Validation of the metabolic score and the new staging system

**Figure 4:**
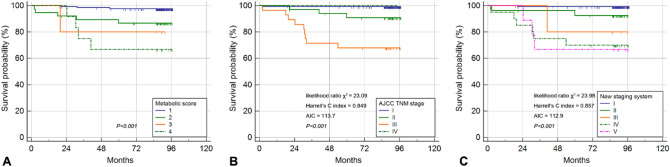
Cumulative DSS curves of the 188 gastric cancer patients according to the metabolic score (**A**), TNM stage (**B**), and new staging system (**C**) in the validation cohort. *DSS* disease-specific survival, *TNM* tumor-node-metastasis.

We performed internal validation of the metabolic score and new staging system established from the training cohort. Figure 4 shows the cumulative DSS curves according to the metabolic score, TNM stage, and the new staging system of the validation cohort. Kaplan–Meier analysis showed that a higher metabolic score was associated with poorer DSS, and the log-rank test showed a significant difference in survival between metabolic scores (*P* < 0.001) (Fig. 4A). The DSS rates were 99.2% in stage I, 91.7% in stage II, 67.9% in stage III, and 100% in stage IV (*P* < 0.001) under the TNM staging system (Fig. 4B). A survivor with stage IV underwent radical gastrectomy with a curative excision of solitary hepatic metastasis. In the new staging system, the DSS rates were 99.2% in category I, 92.6% in category II, 85.7% in category III, 70.0% in category IV, and 66.7% in category V (*P* < 0.001) (Fig. 4C). The χ^2^ likelihood ratio, Harrell’s C index, and AIC value in the new staging system and TNM stage were 23.98 and 23.09, 0.857 and 0.849, and 112.9 and 113.7, respectively, in the validation cohort.

## Discussion

In this study, we developed and internally validated a new risk prediction metabolic score and a new staging system using metabolic parameters of [^18^F]FDG PET/CT for predicting DSS in GC patients who undergo curative surgical resection. First, we developed a metabolic score that combines T_SUVmax and N_SUVmax. Second, because the pT/pN stages and metabolic score were independent prognostic factors for DSS in the multivariate analysis, we developed a new prognostic model by incorporating the metabolic score into the conventional TNM stage for improved DSS prediction in the training cohort. Our new staging system showed better performance for predicting DSS than the conventional TNM stage in the validation and training cohorts.

Several studies have revealed that the degree of [^18^F]FDG uptake by the primary tumor on [^18^F]FDG PET/CT could help predict survival in GC patients^15,16,18,19^. Mochiki et al. demonstrated that those with FDG PET-positive GCs showed significantly lower survival rates than those with FDG PET-negative tumors^16^. Furthermore, among patients who underwent curative surgical resection, those with a higher T_SUVmax had poorer overall survival than those with a lower T_SUVmax^16^. In the metastatic setting, Chung et al. showed that high T_SUVmax was associated with inferior overall survival in patients with metastatic gastric adenocarcinoma^18^. Similarly, Park et al. verified that T_SUVmax was the most robust independent factor for predicting prognosis in stage IV GC patients receiving palliative chemotherapy^19^. Despite the relatively small patient populations of these studies and the different thresholds they proposed, most studies reported that T_SUVmax was a significant prognostic factor for predicting the survival of GC patients possibly because an increased [^18^F]FDG uptake by primary tumors indicates the metabolic status and tumor aggressiveness^20,21^. However, T_SUVmax alone may be inadequate for precise survival prediction in GC patients when N_SUVmax is also considered a prognostic factor. We previously found that the metabolic information of metastatic LNs has a greater prognostic value than that of the primary tumor for predicting the survival of GC patients^17^.

There have been studies on the prognostic value of the metabolic activity of metastatic LNs^15,17,22^. Coupe et al. demonstrated that [^18^F]FDG positivity of LNs and primary tumors was associated with worse overall survival of GC patients^15^. We also found that N_SUVmax was an independent prognostic factor for overall and recurrence-free survival after curative resection in GC patients with LN involvement^17^. More recently, Wang et al. showed that the number of [^18^F]FDG PET-positive LNs could be a useful predictive marker for prognosis in locally advanced GC patients^22^. However, no study has evaluated the prognostic value of the combination of T_SUVmax and N_SUVmax in predicting the survival of GC patients. Notably, the present study showed that a higher metabolic score correlated with poor DSS and was confirmed to be an independent prognostic factor in GC patients.

Two recent studies developed a prognostic model using [^18^F]FDG PET/CT parameters for hepatocellular carcinoma and breast cancer^23,24^; however, a prognostic model using a combination of T_SUVmax and N_SUVmax has not been reported for GC. Accordingly, we developed and internally validated a new staging system that comprises a combination of the metabolic score and the conventional TNM stage, which are independent prognostic factors for DSS in GC patients. Five risk groups were derived from the model, and we found significant differences in DSS among the risk groups. Although direct comparison between the stages of the two models is difficult because of the different subgroups in each stage, the new staging system, which was developed using statistical methods for scientific rationality, showed better discriminatory capability than the conventional TNM staging. In the decision tree, the conventional TNM stage was selected as the first-order risk factor, and patients were divided into two groups (the TNM stage I–II group and the TNM stage III–IV group). The metabolic score was then added as the risk factor for subgroup classification of these two groups. In patients with TNM stage I–II, T_SUVmax could potentially aid in stratifying categories I and II in the new staging system. Furthermore, patients with TNM stage I–II GC and high N_SUVmax were classified into the new staging category III, while all patients with TNM stage III–IV GC were grouped into the new staging category IV–V. All three patients with distant metastasis (TNM stage IV) were also classified into the new staging category V.

Many studies have shown that despite its high prognostic value in many cancers, [^18^F]FDG PET/CT has low sensitivity for detecting LN metastasis^15,17,25^. In the training cohort of the present study, only 59 (10.9%) of 543 patients had a metabolic score of 3 or 4. As such, if only N_SUVmax was used as a prognostic factor, most patients would not be further classified into subgroups. However, considering T_SUVmax as a cooperative prognostic factor, 484 patients with a low N_SUVmax were further divided to have a metabolic score of 1 (n = 359) or 2 (n = 125). This classification using the combination of T_SUVmax and N_SUVmax showed a significant additional effect for the prognostic model as well as independent prognostic value. Although N_SUVmax has a high prognostic value, many patients show negative nodal FDG uptake. Thus, T_SUVmax helps to further group these patients.

The present study had a few limitations. First, this was a single-institution retrospective study that might have been subject to selection bias. For example, all patients who underwent preoperative treatment were excluded because any treatment before surgical resection could affect histopathologic results. Moreover, patients who received delayed surgical treatment (i.e. more than 1 month after [^18^F]FDG PET/CT) were excluded. Second, T_SUVmax in early GC and N_SUVmax in patients with small-sized metastatic LN could have been underestimated due to partial volume effects. Third, we could not completely rule out the possibility of the impact of physiological FDG uptake by the normal stomach wall or increased FDG uptake by inflammatory LNs. Finally, although we internally validated our risk prediction model, further prospective studies and external validation should be conducted to generalize the prognostic impact of the metabolic score and new prognostic model in patients with GC.

In conclusion, this study identified that the metabolic score comprising T_SUVmax and N_SUVmax was an independent predictor of DSS after curative surgical resection in GC patients. Furthermore, the new staging system comprising the metabolic score and TNM stage has superior prognostic performance for risk stratification for DSS than the TNM staging system alone. Therefore, [^18^F]FDG PET/CT could be used not only for individualized preoperative therapeutic planning but also for stratifying patients into different survival groups after surgical treatment and determining appropriate additional treatments.

## Methods

This study followed the medical research protocols and ethical guidelines laid down by the World Medical Association’s Declaration of Helsinki. The Institutional Review Board of Keimyung University Dongsan Hospital approved this retrospective study and waived the requirement to obtain informed consent (2018-06-028).

### Patients

**Figure 5:**
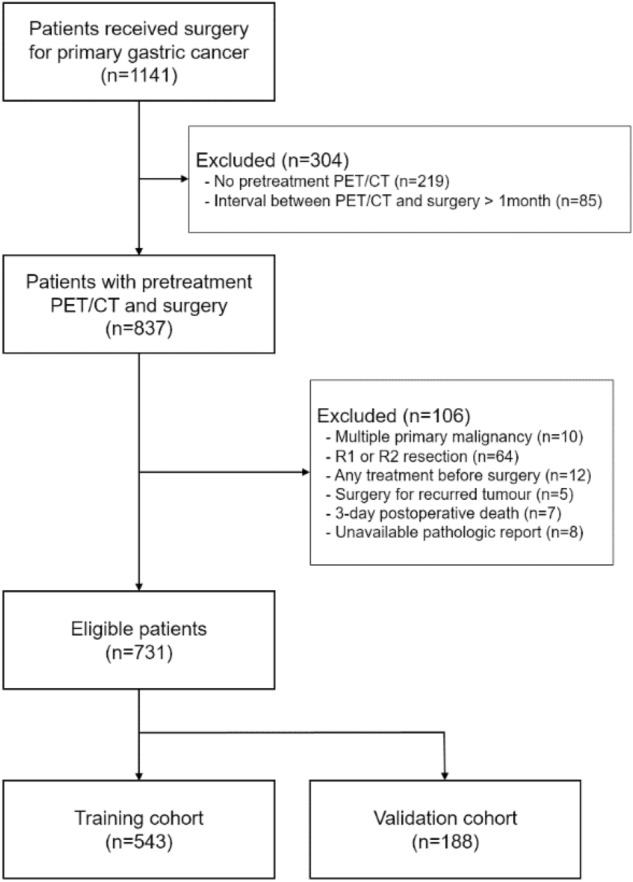
Flow diagram of patient selection.

We retrospectively reviewed medical records of 1141 patients who underwent surgery for primary GC at our institution between January 2008 and December 2011. Of these, 731 patients who underwent preoperative [^18^F]FDG PET/CT for a staging workup and subsequent curative surgical resection were enrolled in this study. The entire cohort was divided into a training cohort (n = 543) that underwent surgery between January 2008 and December 2010, and a validation cohort (n = 188) that underwent surgery between January 2011 and December 2011 (Fig. 5). The exclusion criteria were as follows: multiple primary malignancies, microscopic or macroscopic residual disease after surgical resection, any other treatment before surgery, surgery for GC that recurred, death within 30 days post operation, an unavailable pathologic report, or an interval of more than 1 month between [^18^F]FDG PET/CT and surgery.

All patients underwent subtotal or total gastrectomy along with D2 lymphadenectomy (advanced GC) and D1 + β or D2 lymphadenectomy (early GC). Clinicopathologic data, including sex, age at surgery, tumor location, World Health Organization and Lauren histopathological subtypes, lymphovascular invasion, neural invasion, pT and pN stages were retrieved from patients’ medical records. The survival data was retrieved from the National Health Insurance Service. The pT and pN stages were classified according to the 8th edition of the AJCC TNM staging system.

### [^18^F]FDG PET/CT and image analysis

Before injecting [^18^F]FDG, all patients fasted for at least 6 h, and the blood glucose level was managed to < 150 mg/dL. Patients were instructed to rest during [^18^F]FDG uptake period. Images were acquired 60 min after intravenously administering 5.5 MBq/kg of [^18^F]FDG. [^18^F]FDG PET/CT was performed using 2 integrated PET/CT systems (Discovery STe; GE Healthcare or Biograph mCT; Siemens Healthineers). First, a low-dose CT image (Discovery STe; peak voltage, 120 kV; automated tube current, 60–150 mA; and slice thickness, 3.75 mm, Biograph mCT; peak voltage of 120 kV, automated exposure control using CARE Dose4D, and slice thickness of 3 mm) was acquired for attenuation correction. No oral or intravenous contrast was used. Immediately following CT, PET was performed with an acquisition time of 3 min per bed position with the Discovery STe and 1.5 min per bed position with the Biograph mCT in three-dimensional mode. PET images were reconstructed using an ordered-subset expectation maximum iterative reconstruction algorithm. All 543 training cohort patients underwent PET/CT scan on the Discovery STe. Meanwhile, of 188 validation cohort patients, 82 patients underwent PET/CT scan on the Discovery STe and 106 patients underwent PET/CT scan on the Biograph mCT.

The images were retrospectively interpreted on an AW server 3.2 (GE Healthcare) by two experienced nuclear medicine physicians who were blinded to patient survival outcomes, and a consensus was achieved. First, all [^18^F]FDG PET/CT images were visually assessed and classified as positive or negative with respect to [^18^F]FDG uptake by the primary tumor. Positive uptake was defined as abnormally increased [^18^F]FDG uptake that exceeded the physiologic uptake by the surrounding stomach wall and corresponding cancer lesions on esophagogastroduodenoscopy. Meanwhile, negative uptake was defined as no significantly visible [^18^F]FDG uptake or diffusely increased uptake indistinguishable from physiologic gastric wall uptake. Focally increased [^18^F]FDG uptake lesions that did not correspond to cancer lesions on esophagogastroduodenoscopy and histopathological findings were also judged to be negative [^18^F]FDG uptake. Consequently, T_SUVmax was obtained only in positive [^18^F]FDG uptake lesions. For metastatic LNs, N_SUVmax was acquired in the highest focal [^18^F]FDG-avid LN on the PET image regardless of the size on CT for semiquantitative analysis. Circular regions of interest were manually drawn over the maximum [^18^F]FDG uptake lesions on the attenuation-corrected transaxial [^18^F]FDG PET images. We assigned the SUVmax as 0 to patients with negative [18F]FDG uptake of the primary tumor or LNs. The SUVmax was calculated using the following formula: SUVmax = maximum activity in the region of interest (MBq/g)/(injected dose [MBq]/body weight [g]).

### Statistical analysis

Continuous variables are expressed as means ± standard deviations, and categorical variables as numbers and percentages. The metabolic score, which is a combined index of T_SUVmax and N_SUVmax, was developed for predicting DSS using CTree analysis using the R package “party”^26^. DSS was defined as the interval between surgery and date of cancer-specific death (deaths from other causes were censored) and was calculated using the Kaplan–Meier method. Multivariate Cox proportional hazards regression analyses were performed to identify independent variables affecting DSS, and the HRs and 95% CIs were estimated for each parameter. The new staging system, which was a tree-structured survival model created using CTree analysis, was established by combining the metabolic score and TNM stage in the training cohort.

The prognostic performance of the new staging system and TNM stage were statistically assessed. To compare the homogeneity of the TNM stage with that of the new staging system, the χ^2^ likelihood ratio test related to the Cox regression model was used. The discriminatory capability of gradient assessments was evaluated using the AIC and Harrell’s C index. Models with higher χ^2^ likelihood ratios and Harrell’s C indices were deemed accurate. In addition, a lower AIC value indicates that the model attains a better balance between the overall fit to the data and the model’s simplicity^27,28^. To determine generalizability of the established metabolic score and new staging system derived from the training cohort for a risk prediction model, internal validation was performed in the validation cohort. All statistical analyses were performed using MedCalc for Windows, version 18.6 (MedCalc Software), and R version 3.4.3 software (http://www.r-project.org, R Foundation for Statistical Computing). *P* values < 0.05 were considered statistically significant.

## Data Availability

The datasets generated and/or analyzed during the current study are available from the corresponding author on reasonable request.
